# Effects of p35 Mutations Associated with Mental Retardation on the Cellular Function of p35-CDK5

**DOI:** 10.1371/journal.pone.0140821

**Published:** 2015-10-15

**Authors:** Shunsuke Takada, Keiko Mizuno, Taro Saito, Akiko Asada, Karl Peter Giese, Shin-ichi Hisanaga

**Affiliations:** 1 Laboratory of Molecular Neuroscience, Department of Biological Sciences, Graduate School of Science, Tokyo Metropolitan University, Minami-Osawa, Hachioji, Tokyo, Japan; 2 Centre for Cellular Basis of Behavior, Institute of Psychiatry, King's College London, 125 Coldharbour Lane, London, United Kingdom; McGill University Department of Neurology and Neurosurgery, CANADA

## Abstract

p35 is an activation subunit of the cyclin-dependent kinase 5 (CDK5), which is a Ser/Thr kinase that is expressed predominantly in neurons. Disruption of the *CDK5* or *p35* (*CDK5R1*) genes induces abnormal neuronal layering in various regions of the mouse brain via impaired neuronal migration, which may be relevant for mental retardation in humans. Accordingly, mutations in the *p35* gene were reported in patients with nonsyndromic mental retardation; however, their effect on the biochemical function of p35 has not been examined. Here, we studied the biochemical effect of mutant p35 on its known properties, i.e., stability, CDK5 activation, and cellular localization, using heterologous expression in cultured cells. We also examined the effect of the mutations on axon elongation in cultured primary neurons and migration of newborn neurons in embryonic brains. However, we did not detect any significant differences in the effects of the mutant forms of p35 compared with wild-type p35. Therefore, we conclude that these p35 mutations are unlikely to cause mental retardation.

## Introduction

Cyclin-dependent kinases (CDKs) are a family of Ser/Thr kinases that are activated by binding to a regulatory subunit called cyclin and function in cell-cycle progression in proliferating cells [1]. CDK5 is a unique CDK that is expressed in postmitotic neurons [2, 3]. CDK5 is activated by binding to one of its activation subunits, p35 or p39, and plays an important role in various neuronal functions, including neuronal survival, neuronal migration, neurite elongation, and synaptic activity [4–7]. Mice with a deletion of Cdk5 exhibit perinatal lethality and abnormal neuronal cell layers in various brain areas, including the cerebral cortex [4, 8]. Recently, a loss-of-function mutation of CDK5 was reported in human lissencephaly [9]. In contrast to what was observed for CDK5, mice without p35 survive and are fertile, but display inverted neuronal cell layers in the cerebral cortex [10]. However, the milder developmental defects caused by a loss-of-function mutation of p35 are likely to be associated with psychiatric disorders. Conversely, the abnormal activation of CDK5 induced by cleavage of p35 to p25 has been implicated in neurodegenerative diseases [11], although such a gain-of-function mutation of p35 has not been reported.

Mutations of the *p35* gene have been reported in the coding and 3′-untranslated region (UTR) in patients with nonsyndromic mental retardation [12]. The mutations in the coding sequence are two missense mutations, Ala108 to Val (c.323C>T) [A108V] and Leu302 to Ile (c.904C>A) [L302I]; furthermore, one nonsense mutation, Leu178Leu (c.532C>T), was found in a patient who carried the A108V mutation [12]. These mutations are present in the p25 C-terminal region, which includes the CDK5 activation domain. In particular, the A108V mutation is located relatively close to the calpain cleavage site at Phe98/Ala99 [13]. p25 production has been suggested to be involved not only in neurodegenerative diseases, but also in physiological synaptic activity [14, 15]. It is important to examine whether the mutations identified affect the properties of p35-activated CDK5.

In this study, we examined the effect of p35 mutations on the properties of p35-CDK5 using overexpression in cultured cell lines, primary neurons and neurons in embryonic mouse brains.

## Materials and Methods

### Ethics Statement

All animal experiments were performed according to the guidelines for animal experimentation of Tokyo Metropolitan University. The study was approved by the Research Ethics Committee of Tokyo Metropolitan University (approval number, A27-4). All efforts were made to reduce the suffering of animals used.

### Antibodies and chemicals

The anti-myc antibody (4A6) was obtained from Millipore (Billerica, MA). The anti-CDK5 antibody (C-8) was purchased from Santa Cruz Biotechnology (Santa Cruz, CA). The anti-actin antibody was purchased from Sigma (St Louis, MO). Horseradish peroxidase (HRP)-conjugated goat anti-mouse IgG and HRP-conjugated swine anti-rabbit IgG were purchased from Dako (Glostrup, Denmark). Alexa Fluor 488-conjugated anti-mouse IgG was purchased from Invitrogen (Carlsbad, CA). 4′,6-Diamidino-2-phenylindole (DAPI) was from Dojindo (Kumamoto, Japan). Phos-tag acrylamide was obtained from Wako (Osaka, Japan). Protein A/G Sepharose was purchased from GE Healthcare (Buckinghamshire, UK).

### Construction of p35 mutant expression plasmids

pcDNA3-human p35-myc, pCMV5-human CDK5, and the kinase-negative (kn) mutant of CDK5 (D144N) were described previously [16]. The p35-myc A108V or L302I mutant of p35 was constructed by polymerase chain reaction (PCR) using pcDNA3-human p35-myc as a template and 5′–CCCAGCCGCCTGTACCCCCGGCCAG–3′ and 5′–CAAGAAGCGGCTCATCCTAGGCCTGG–3′ as forward and reverse primers, respectively, for A108V; or 5′–CTGGCCGGGGGTACAGGCGGCTGGG–3′ and 5′–CCAGGCCTAGGATGAGCCGCTTCTTG–3′ as forward and reverse primers, respectively, for L302I. The A108V:L178L mutant of p35 was constructed by PCR using pcDNA3-human p35-myc A108V as a template and 5′–CCCGTGCTCTGGTTGCGCAGCGTG–3′ and 5′–CACGCTGCGCAACCAGAGCACGGG–3′ as forward and reverse primers, respectively. Mutations were confirmed by DNA sequencing.

### Cell culture and transfection

COS-7 cells were cultured in Dulbecco’s modified Eagle’s medium (KOHJIN BIO, Saitama, Japan) containing 8% fetal bovine serum (JRH, Lenexa, KS), and transfected with the indicated plasmids using Lipofectamine 2000 (Invitrogen) according to the manufacturer’s protocol. COS-7 cells expressing p35 or its mutants were treated 24 h after transfection with 20 μM ionomycin for 24 h or 50 μg/mL of cycloheximide (CHX) for 1, 6, or 24 h [17].

ICR mice were obtained from Sankyo Laboratory Service (Tokyo, Japan). Mice were housed in a temperature-controlled room under a 12 h light/dark cycle with ad libitum access to food and water. All experiments were performed according to the guidelines for animal experimentation of Tokyo Metropolitan University (Tokyo, Japan). Brain cortices from mice at embryonic day 15 (E15) were dissected and plated on polyethyleneimine-coated glass coverslips at a density of 2.0 x 10^5^ neurons/cm^2^ as described previously [18]. Primary neurons were transfected with the indicated plasmids using Lipofectamine 2000 (2 μg of plasmid DNA mixed with 4 μL of the reagent per well of 12 well plate, (Thermo Fisher Scientific, Waltham, MA). The medium was changed to Neurobasal medium 4 h after transfection. The length of axons was measured at 3 DIV using ZEN imaging software (Carl Zeiss, Jena, Germany).

### Immunoprecipitation and kinase assay of p35-CDK5

COS-7 cells expressing CDK5 and p35 or its mutants were lysed in 20 mM MOPS, pH 6.8, 1 mM EGTA, 0.1 mM EDTA, 0.3 M NaCl, 1 mM MgCl_2_, 0.5% Nonidet P-40, 10 μg/mL leupeptin, 0.4 mM Pefabloc SC and 1 mM dithiothreitol. The cell lysates were centrifuged at 18,000 × g for 30 min at 4°C. The supernatants were incubated with the anti-CDK5 antibody C8 for 1 h at 4°C, followed by incubation with Protein A/G Plus-Agarose for 1 h at 4°C. Agarose beads were washed three times with washing buffer (10 mM MOPS, pH 6.8, 0.5 mM EGTA, 0.05 mM EDTA, 0.15 M NaCl, 0.5 mM MgCl_2_, and 0.25% Nonidet P-40) and then washed with kinase assay buffer (10 mM MOPS, pH6.8, 0.1 mM EGTA, 0.1 mM EDTA, and 1 mM MgCl_2_). The kinase activity of CDK5 was measured using 0.1 mg/mL of histone H1 as a substrate in the presence of 0.1 mM [γ-^32^P] ATP at 35°C for 1 h [19]. The reaction was stopped by boiling in Laemmli’s sample buffer for 3 min. The radioactivity incorporated into histone H1 was measured using a FLA7000 Bioimage analyzer (GE Healthcare) after sodium dodecyl sulfate–polyacrylamide gel electrophoresis (SDS–PAGE).

### Immunofluorescence observation

COS-7 cells expressing CDK5 and p35 or its mutants were cultured on cover slips and fixed with 4% paraformaldehyde in phosphate-buffered saline (PBS) for 30 min at room temperature, which was followed by incubation with an anti-myc (1:1000) antibody in PBS containing 5% normal goat serum and 0.1% Triton X-100 for 1 h and a second incubation with a secondary antibody conjugated to Alexa Fluor 488 (1:500) [20]. Nuclei were stained with DAPI. Cells were examined under a LSM 5 EXCITER confocal microscope (Carl Zeiss) or a fluorescence microscope (BZ-X700; Keyence, Osaka, Japan).

### *In utero* electroporation

*In utero* electroporation was performed as described previously [21]. Plasmid vectors were injected into embryonic mouse brains at E14 and electroporated *in utero*. Mice were sacrificed at E18 and brains were fixed for 24 h at 4°C in 4% paraformaldehyde. Brains were sliced and observed using a macro zoom microscope MVX-10 (Olympus, Tokyo, Japan). GFP-positive neurons were counted using Image J software.

### SDS–PAGE, Phos-tag SDS–PAGE, and immunoblotting

SDS–PAGE was carried out on a 12.5% polyacrylamide gel and Phos-tag SDS–PAGE was performed on a 7.5% acrylamide gel containing 50 μM Phos-tag and 100 μM MnCl_2_, as described previously [22,23]. Membranes were probed with an anti-myc antibody for myc-tagged p35 (1:5000) or an anti-actin antibody (1:1000) followed by an HRP-conjugated secondary antibody. Immunodetection was carried out using an ECL system (GE Healthcare) or the Millipore Immobilon Western chemiluminescent HRP substrate (Millipore).

## Results

### Expression levels of p35 mutants and cleavage to p25

**Fig 1:**
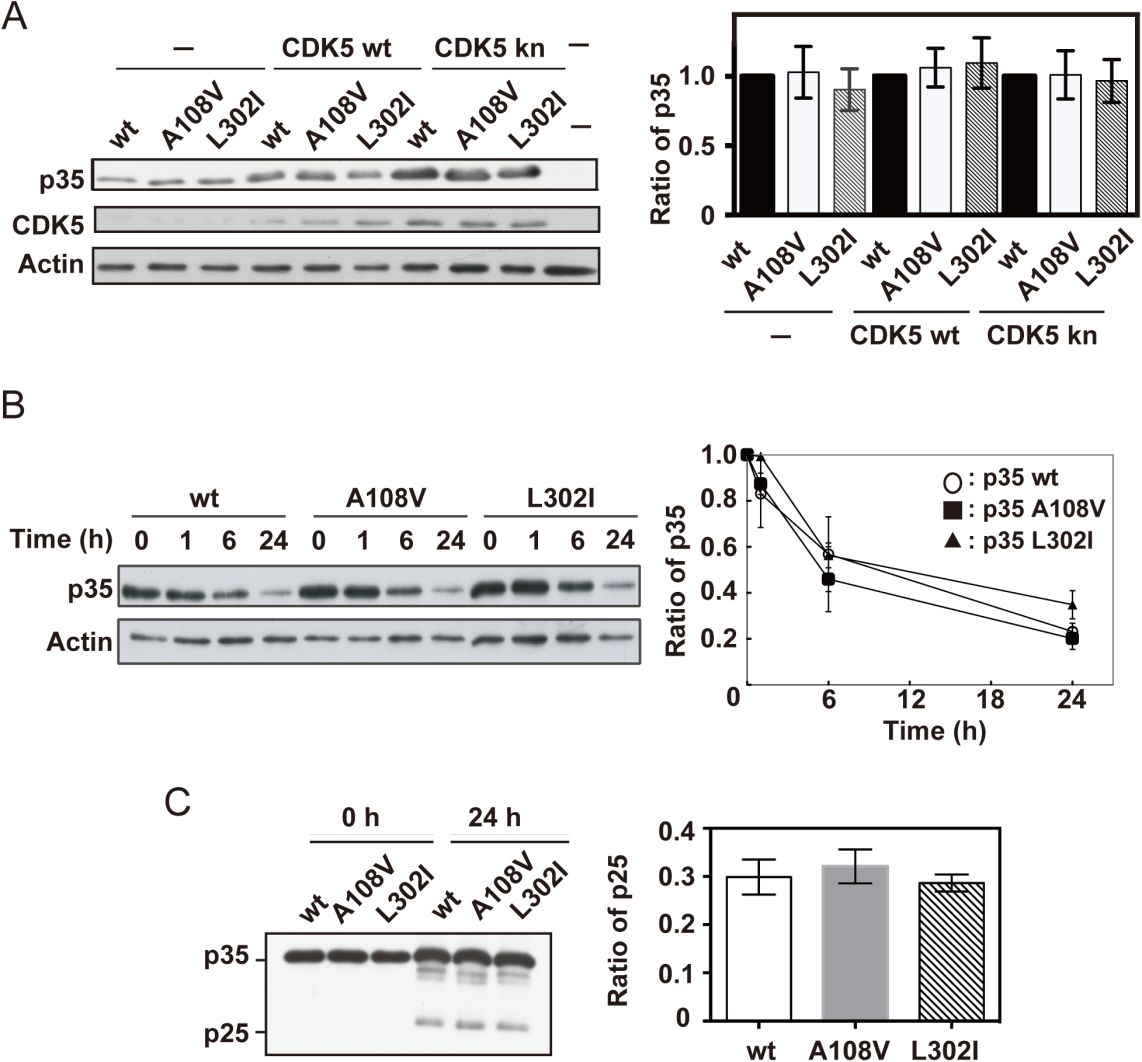
Degradation and cleavage of p35 and its mutants. (A) Expression of p35 in COS-7 cells. Wild-type p35 (wt) or its mutants, p35 A108V:L178L (A108V) and p35 L302I, were expressed alone (–) or together with wild-type (wt) CDK5 or kinase-negative (kn) CDK5 D144N in COS-7 cells. p35 was detected by immunoblotting using an anti-myc antibody. Quantification is shown in the lower panel (means ± SEM, *n* = 3, one-way ANOVA). (B) COS-7 cells expressing p35 wt, A108V or L302I mutant were treated with 50 μg/mL of cycloheximide (CHX) for the indicated times. p35 and its mutants were detected by immunoblotting using an anti-myc antibody. Quantification is shown in the lower panel (means ± SEM, *n* = 3, one-way ANOVA). (C) Cleavage of p35 to p25 in COS-7 cells. COS-7 cells transfected with p35 and knCDK5 were treated with 20 mM ionomycin for the indicated times. p35 and p25 were detected by immunoblotting using an anti-myc antibody. p25 production is quantified and the ratio of p25/p35 is shown on the right (means ± SEM, *n* = 3, one-way ANOVA).

p35 is a protein with a short half-life, and its levels are regulated by proteasomal degradation [13,24]. First, we examined the expression levels of the p35 mutants A108V:L178L (referred to as A108V hereafter) and L302I in COS-7 cells. The expression of wild-type (wt) p35 or its mutants alone (–) or of either CDK5 or kn CDK5 D144N led to the observation of similar expression levels of p35 in the wt and mutants (Fig 1A). The degradation rate of p35 was compared in the presence of CHX for 1, 6, and 24 h. There was no clear difference in the decrease of p35 between the wt and mutants (Fig 1B).

p35 is cleaved by calpain between Phe98 and Ala99, to produce the stable C-terminal fragment p25 [25–27]. The A108V mutation is present at +9 amino acids from the cleavage site. We examined if these mutations influence the cleavage of p35 to p25. We coexpressed p35 (wt, A108V, or L302I) with knCDK5 in COS-7 cells, and treated the cells with 20 μM ionomycin for 24 h, to activate calpain [17]. Here, we used knCDK5 because CDK5 activity inhibits the cleavage of p35 via phosphorylation at Thr138 [28]. About 20% of p35 wt was cleaved to p25 after treatment with ionomycin for 24 h. However, there was no difference in p25 generation between the wt and mutants (Fig 1C).

A theoretically possible effect of the p35 mutations is on the interaction of Cdk5 with the activator p39. However, we consider this as unlikely because the p35 mutations did not affect the expression level of p35. Further, Cdk5 is expressed at much higher levels than its activators in neurons [3]. Thus, the interaction between p39 and Cdk5 should be affected only if the expression of p35 were increased remarkably. Therefore, we focused on p35 alone in the following experiments.

### Binding of p35 to CDK5 and kinase activity of p35-CDK5

**Fig 2:**
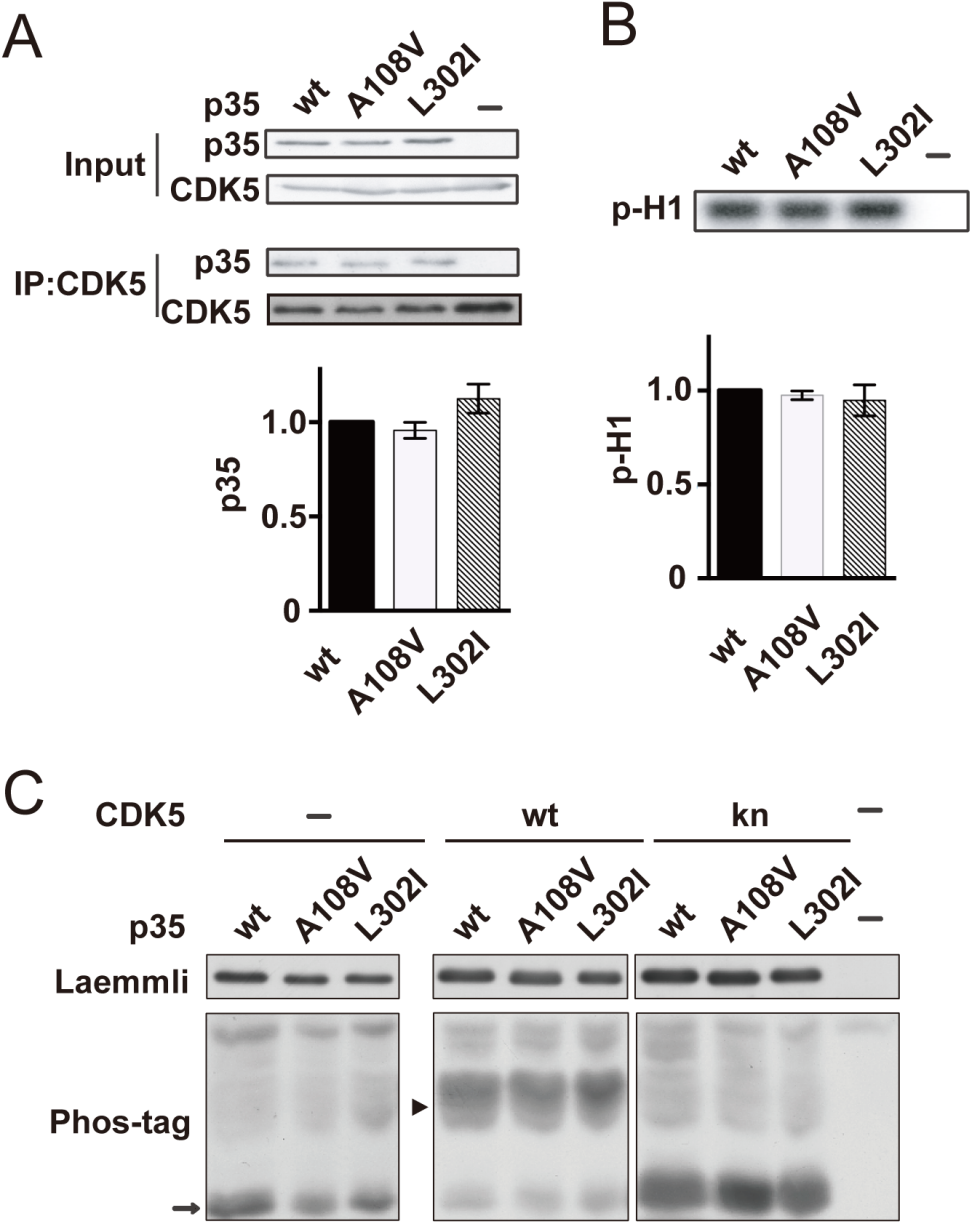
Activation of CDK5 by p35 or its mutants. (A) The binding of p35 to CDK5. Wild-type p35 (wt), p35 A108V:L178L (A108V), or p35 L302I was coexpressed with CDK5 in COS-7 cells. CDK5-p35 was immunoprecipitated from the cell lysates using the anti-CDK5 antibody. Quantification is shown at the bottom (means ± SEM, *n* = 3, one-way ANOVA). (B) The kinase activity of CDK5 complexed with p35 or its mutants. p35 wt or its mutant, A108V or L302I, was transfected into COS-7 cells together with CDK5. Kinase activity was measured based on CDK5-p35 immunoprecipitated from COS-7 cells using an anti-CDK5 antibody. Histone H1 kinase activity was measured in the presence of [γ-^32^P] ATP for 1 h at 35°C. The upper panel shows an autoradiograph and the lower panel shows the quantification (means ± SEM, *n* = 3, one-way ANOVA). (C) Phos-tag SDS–PAGE of p35 or its mutant expressed without (–) or with CDK5 or knCDK5. p35 or its mutant, A108V or L302I, was expressed alone (–) or with CDK5 or knCDK5 in COS-7 cells. Laemmli’s SDS–PAGE and Phos-tag SDS–PAGE are shown in the upper and lower panels, respectively. p35 was detected by immunoblotting using an anti-myc antibody. Nonphosphorylated and phosphorylated p35 are shown by the arrow and arrowhead, respectively.

Next, we examined whether mutations alter the binding or activation ability of p35 to CDK5. CDK5 was immunoprecipitated from the lysates of cells expressing CDK5 and p35 wt or its mutants, and the amount of p35 bound to CDK5 was estimated by immunoblotting, whereas kinase activity was assessed by histone H1 phosphorylation (Fig 2A and 2B). There was no difference in p35 binding to CDK5 or in histone H1 phosphorylation by CDK5 activated by p35 wt or mutants. These results indicate that these mutations do not change the kinase activity of CDK5 *in vitro*. We assessed the CDK5 activity in cells by phosphorylation of p35 at Ser8 [23]. Phosphorylation of Ser8 represents the kinase activity of the p35-CDK5 complex in cells [16]. We compared Ser8 phosphorylation using Phos-tag SDS–PAGE [23]. Nonphosphorylated p35 expressed alone or together with knCDK5 showed faster migration on Phos-tag gels (arrows in Fig 2C), whereas p35 with phosphorylation at Ser8 exhibited slower mobility (arrowhead in Fig 2C). No clear difference was found in the banding patterns between p35 wt and its mutants (Fig 2C).

### Subcellular distribution of p35 in cultured cell lines

**Fig 3:**
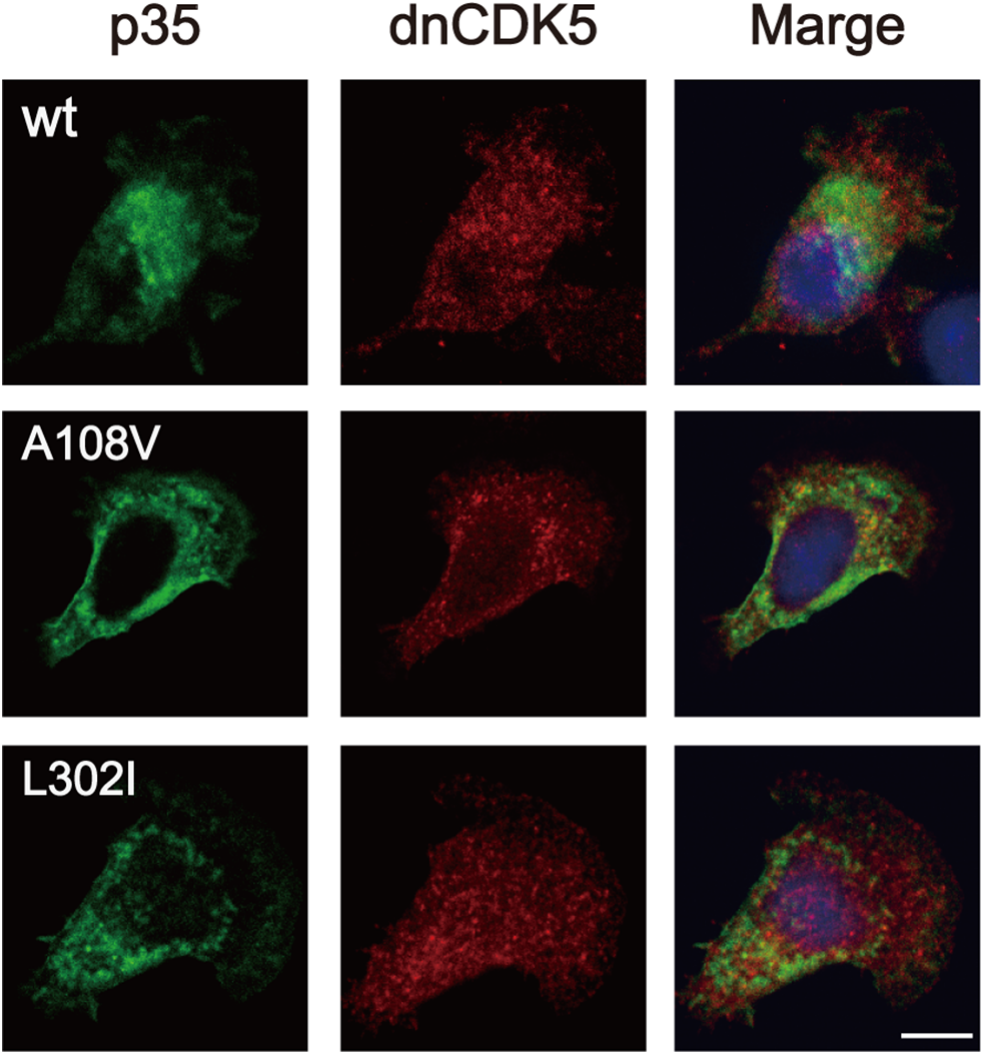
Subcellular localization of p35 or its mutants in Neuro2A cells. p35, wt, A108V or L302I, were coexpressed with knCDK5 in Neuro2A cells. Neuro2A cells were immunostained with an anti-myc antibody for p35 (green) and anti-CDK5 (red) at 24 h after transfection. Merges are shown in right panels. The blue signals indicate nuclei stained with DAPI. Scale bar, 10 m.

p35-CDK5 is a membrane-bound kinase. We studied the effect of these mutations on the subcellular distribution of p35 in Neuro2A and COS-7 cells. We transfected p35 (wt, A108V, or L302I) together with knCDK5 in these cells, and observed the subcellular distribution of p35 by immunofluorescent staining. Since the similar results were obtained, the results of Neuro2A cells are shown in Fig 3. When p35 was coexpressed with knCDK5, p35 mutants showed perinuclear and plasma-membrane localization, as did wt p35 (Fig 3). We did not observe a clear difference in the localization of p35 between the wt and mutants (Fig 3).

### The effect of p35 mutation on axon outgrowth

**Fig 4:**
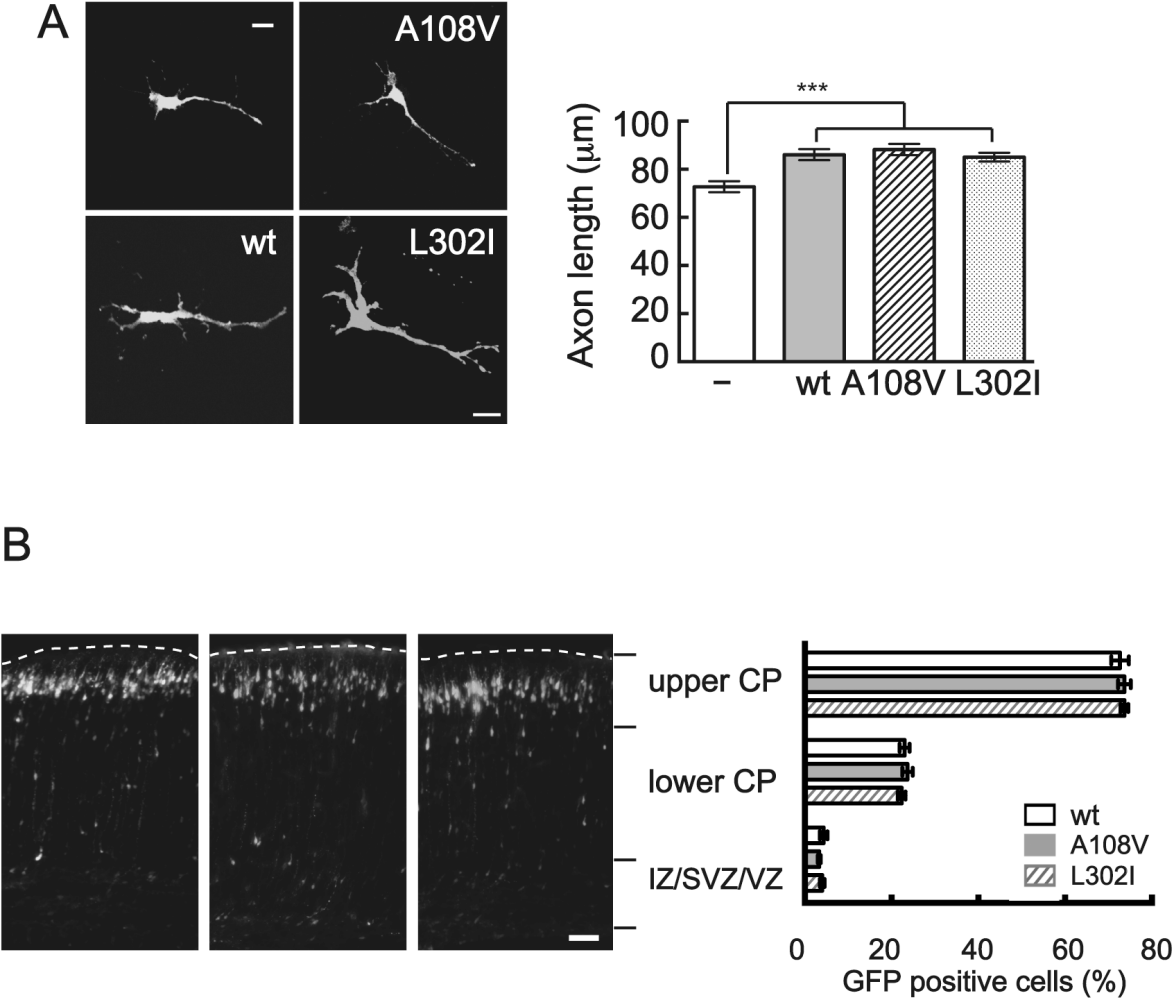
Effect of p35 mutations on axon elongation and neuronal migration. (A) Mouse brain cortical neurons in culture were transfected with p35, wt, A108V or L302I, with EGFP using Lipofectoamine 2000 at 0 DIV. Axons were observed with EGFP and measured at 3 DIV, using Zen software associated with confocal microscopy. Quantification is shown on the right side (mean ± SEM, n = 50 for control, 50 for p35, 50 for p35 A108V, and 50 for p35 L302I, ***, P < 0.001, One way Anova). Scale bar, 20 μm. (B) p35 or its mutants were transfected into neurons with EGFP by *in utero* electroporation at E14 and positions of neurons were observed with EGFP at E18. Positions of upper cortical plate (CP), lower CP and Intermediate/subventricular/ventricular zones are indicated at the right side of fluorescent micrographs. Quantification is shown in right (means ± SEM, n = 200 ~ 250 neurons from 3 embryonic brains each of wt, A108V and L302I). Scale bar, 50 μm.

It is known that CDK5-p35 regulates axon outgrowth. We addressed functional effects of p35 mutations on axon outgrowth of primary cortical neurons. Overexpression of p35 increased the length of axons (Fig 4A), consistent with previous reports that CDK5-p35 positively regulates axon elongation [29, 30]. However, there were no differences in axon length between p35 wt and its mutants (Fig 4A).

We also tested the effect of p35 mutations on migration of newborn neurons in mouse embryonic brains, using *in utero* electroporation. Neurons were labeled with EGFP, which was co-introduced with p35 or its mutants at E14. Most of EGFP-positive neurons migrated to the outer cortical layer when observed at E18 and no difference was detected between the wt and mutants (Fig 4B).

## Discussion

Missense mutations in p35 were reported in patients with sporadic mental retardation [12]. In this study, we examined the effect of mutations on several known properties of p35 by expressing the mutants in cultured cells or neurons together with CDK5. However, these p35 mutants showed no differences in half-life, cleavage by calpain, CDK5 binding and activation, cellular localization, axon outgrowth and neuronal migration, compared with wt p35. These mutations may not be disease-causing mutations, although we cannot exclude the possibility that they influence as yet uncharacterized properties of p35.

**Fig 5:**
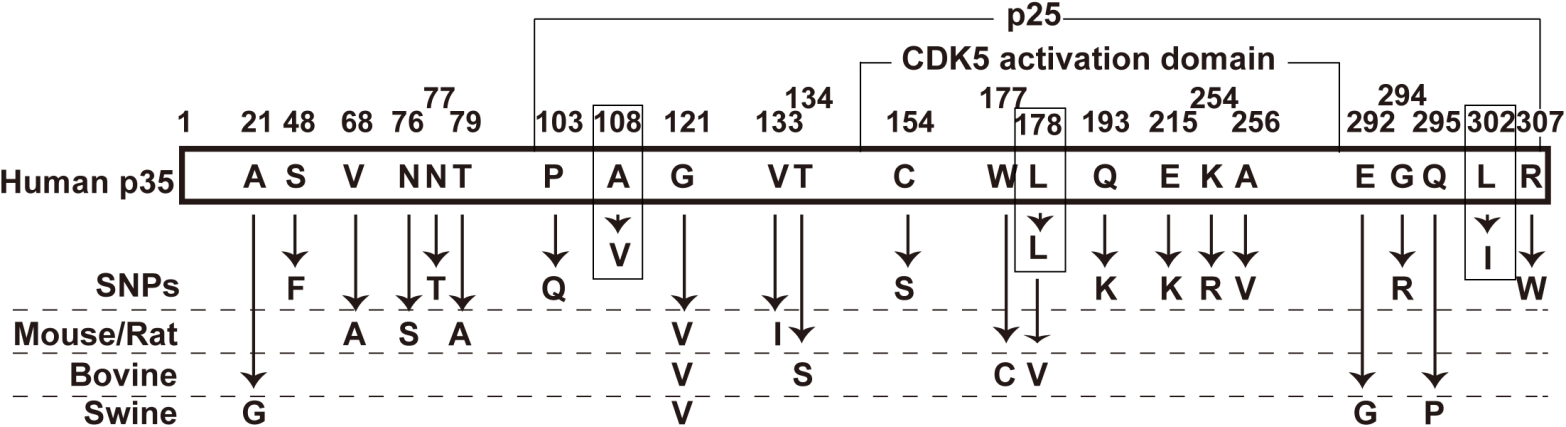
SNPs located in human p35 and amino acid differences between human and other mammalian p35 forms. Amino acid exchanges reported as SNPs for human p35 or for other mammalian p35 forms. A108A and L302L are indicated by a box. The p25 and CDK5-activation domains are indicated above the molecule.

The mutations in question are Ala108 and Leu302, located outside the minimum CDK5 activation domain (amino acids 147 to 292) in the C-terminal p25 region (Fig 5, box). According to the crystal structure of CDK5-p25 [31], these sites are located in flexible regions, where the positions of amino acids have not been determined. Ala108 may be located in the linker region that connects the N-terminal p10 and CDK5 activation domains, each of which has distinct functions in the cellular localization and activation of CDK5, respectively. The linker region includes the calpain-cleavage site (at Phe98 and Ala99) [25–27]. A recent report showed that the deletion of the six N-terminal amino acids, with replacement of Ala with Leu at the cleavage site, confers resistance to calpain cleavage to p35 [15]. This suggests that the N-terminal sequence is more important for recognition by calpain. Ala108 is located 10 amino acids downstream of the cleavage site; therefore, the mutation might not affect cleavage.

Leu302 is located in the C-terminal end of p35, which is dispensable for CDK5 binding and activation [32, 33]. The role of this region in other properties of p35 has not been addressed. The inspection of the sequence surrounding Leu302 revealed that the ^297^DKKRL**L**L^303^ sequence matches the classical di-leucine motif of (D/E)xxxL(L/I), which determines the endosomal or lysosomal trafficking of transmembrane proteins [34]. Although p35 contains triple-leucine, but not di-leucine motifs, the C-terminal LLL sequence has been shown to function as the di-leucine signal for the plasma membrane Ca^2+^ ATPase 4b (PMCA4b) [35]. Although p35 is not a transmembrane protein, it associates with membranes via N-terminal myristoylation [20, 25, 36]. It is possible that the sequence may regulate the localization of p35-CDK5 to particular membrane compartments [37]. Even if this is true, the L302I mutation might not change the distribution of p35-CDK5 at the perinuclear region in cultured cells (Fig 3) because isoleucine is an amino acid that can be exchanged with leucine in the di-leucine motif. Whether this sequence functions as an endosomal-localization signal represents a question to be addressed in future studies.

Impairment of neuronal migration during brain development may be a cause of mental retardation [38]. CDK5 is a factor that regulates neuronal migration and axon elongation [4, 7, 10], and in developing brains the kinase activity of CDK5 is mainly activated by p35, but not p39 [39, 40]. Therefore, we examined the effect of mutations on axon outgrowth and neuronal migration, impairments of which are thought to contribute to mental retardation. However, we neither detected any differences between the wt and its mutants in both axon outgrowth and neuronal migration.

p35 is a conservative protein that is 100% identical in amino acid sequence between the mouse and the rat, and 98.4% identical between humans and the mouse/rat sequence. The amino acid differences in p35 between several mammalian species and humans are shown in Fig 5. The differences are distributed throughout the molecule. Interestingly, with the exception of the Gly-to-Val exchange at amino acid 121, differences were detected in distinct regions of p35 in a species-dependent manner: the N-terminal region in the mouse and rat, the CDK5-binding domain in bovine, and in the C-terminal-most region in porcine, with no consistent replacement. The L178L mutation is the site at which Leu is replaced with Val in bovine p35 (Fig 5, box). Ten single-nucleotide polymorphisms (SNPs) located in the coding region of human p35 were found in PubMed (http://www.ncbi.nlm.nih.gov/snp/?term=Cdk5r1+human) (Fig 5, SNPs). These SNPs are also distributed throughout the molecule, with a few SNPs being located in the α-helical region of the CDK5-activation domain. A comparison of the mutations with these SNPs did not reveal any particular differences between them. Taken together, our results suggest that the A108V and L302I mutations are conservative amino acid exchanges that do not appear to alter p35 function.

In this study, we focused on the missense mutations found in the coding region of p35 (*CDK5R1)* gene. However, Venturin et al. (2006) also reported several mutations in the 3’-UTR region. The 3’-UTR includes DNA sequences involved in post-transcriptional regulation of gene expression such as the stability and translational efficiency of mRNA. Several 3’-UTR sequence-binding proteins are recently reported for p35 mRNA [41]. However, the effect of mutations in 3’-UTR region on the stability and transcriptional activity has not been examined yet. This will be a future question to be answered.
